# Supplementary material for: Detailed modeling of positive selection improves detection of cancer driver genes
Source: Nat Commun. 2019 Jul 30;10:3399. doi: 10.1038/s41467-019-11284-9 (PMC6667447; doi:10.1038/s41467-019-11284-9)
Supplement: Supplementary file 1 — Supplementary Information [file 41467_2019_11284_MOESM1_ESM.pdf]

Supplementary Information

Detailed modeling of positive selection significantly improves detection of cancer driver genes

Zhao et al

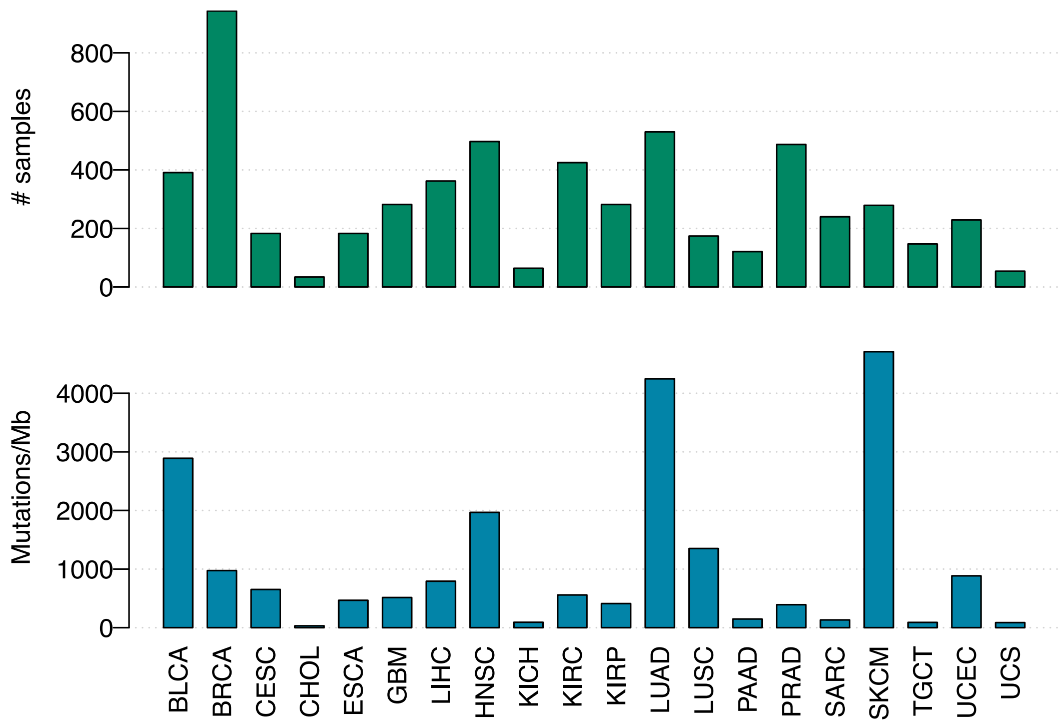

Supplementary Figure 1. Cohort sizes and mutation rates for 20 tumor types analyzed in this study.
Top panel, numbers of tumor samples in each of the 20 tumor cohorts. Bottom panel, mutation rates in each of the 20 tumor cohorts. Here, mutation rate in each cohort is calculated as the total number of mutations across all the samples in that cohort per Megabase (Mb).

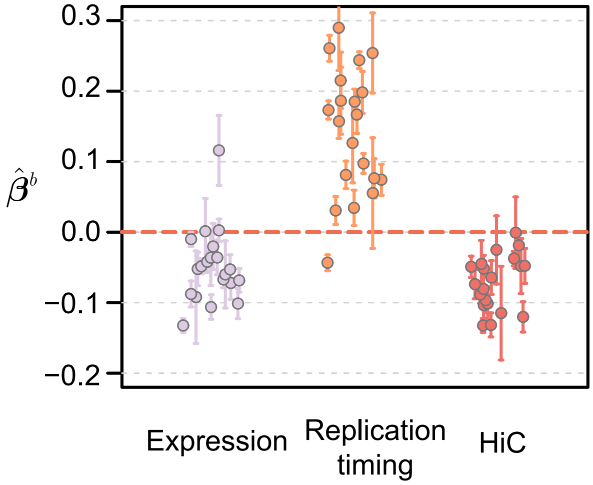

Supplementary Figure 2. Estimated effect sizes for three background features.
Each dot represents an estimate from one tumor type with error bars representing ± standard error. Expression: normalized RNA sequencing gene level RSEM values; replication timing, DNA replication time of this gene (measured in HeLa cells); HiC, measured from HiC experments in K562 cells. All three features are scaled to have mean of 0 and standard deviation of 1.

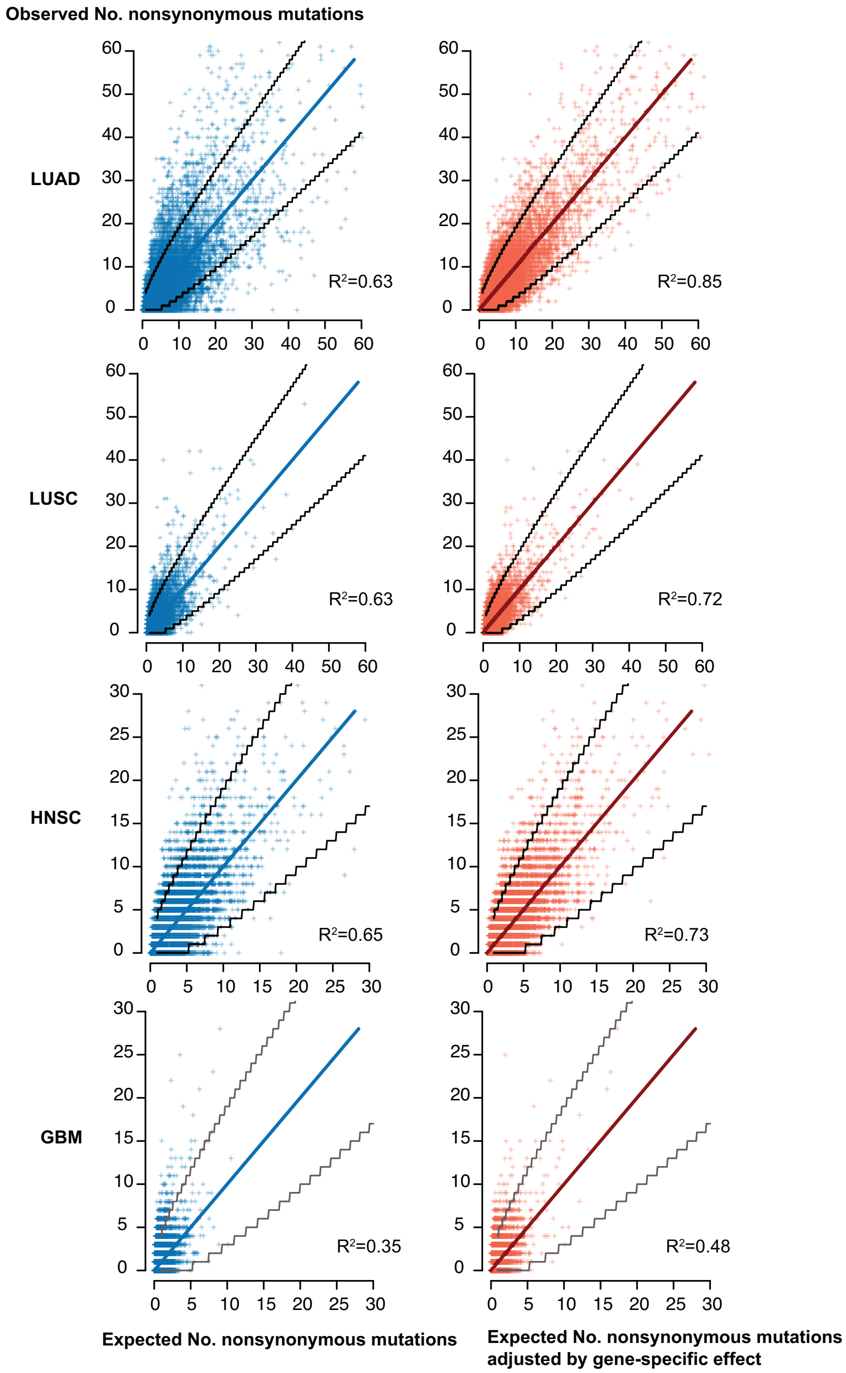

Supplementary Figure 3. Effects of adjustments of local variation

4 representative tumor types LUAD, LUSC, HNSC and GBM are chosen to show the effect of adjustment of local variation. In each row, scatterplots of expected number of nonsynonymous mutation in each gene with (left) and without (right) local variation adjustment versus the observed number are displayed for one tumor type. The adjustment is calculated as the posterior mean of λ fitting synonymous mutation data. Grey lines indicate upper and lower bounds of 99% confidence interval based on Poisson test given expected rate. The diagonal line has slope =1 and R^2 was calculated using this as the regression line.

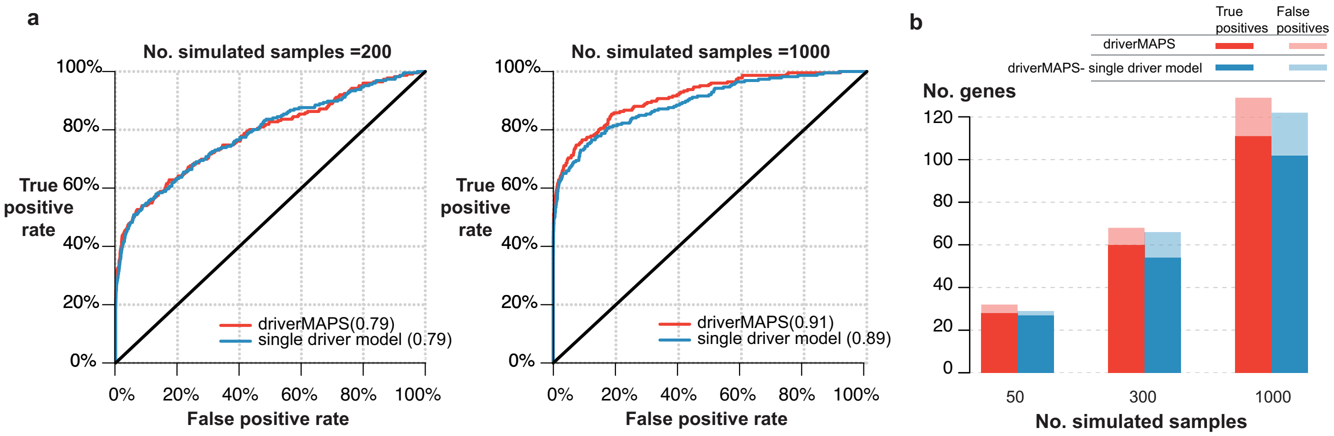

Supplementary Figure 4. Performance evaluation for "single driver" model of driverMAPS using simulations

For the "single driver" model, we pooled all training genes into one set, without distinguishing OGs and TSGs, and estimated parameters for the selection model (including effect sizes for functional features and parameters for modeling spatial effect). We then use this selection model as the alternative model and calculate Bayes factors the same way as in driverMAPS. We used the same data here as used in Figure 3b-d and also the same evaluation procedures. **(a)** ROC curves comparing driverMAPS (TSG/OG separate modeling) with "single driver" model version of driverMAPS, at sample sizes 200 and 1000. **(b)** Number of true positive and false positive genes at FDR<0.1 for driverMAPS and its" single driver" model version.

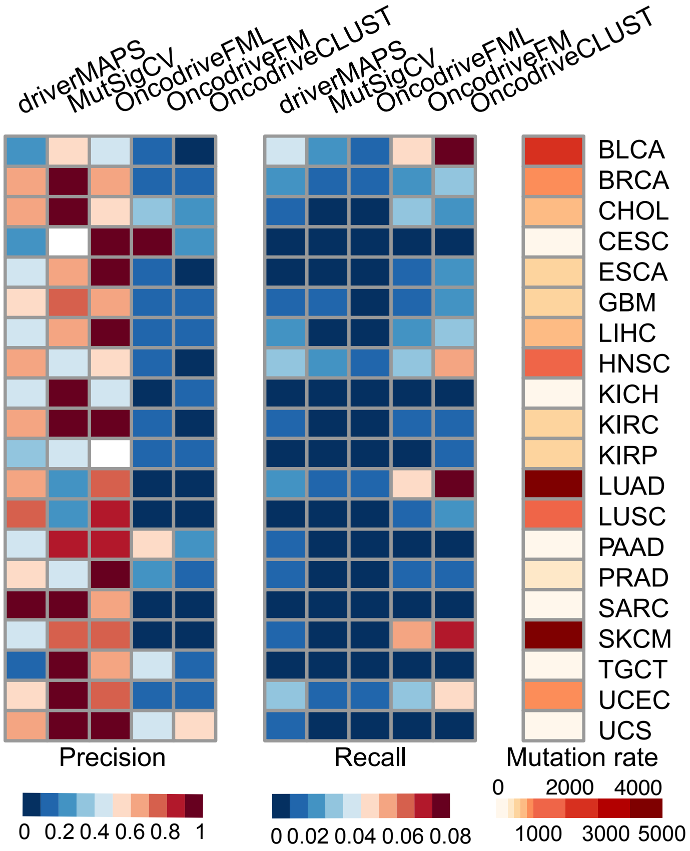

Supplementary Figure 5. Effects of mutation rate on precision and recall rate for different methods.
Values for precision, recall and mutation rate for each tumor type are shown in heatmaps. We calculated precision (proportion of known cancer genes among significant genes) and recall rate (proportion of known cancer genes found significant among all known cancer genes) for each tumor type with significance cutoff at FDR =0.1. We calculated mutation rates for each cancer type as number of mutations per Mb summing across all samples.

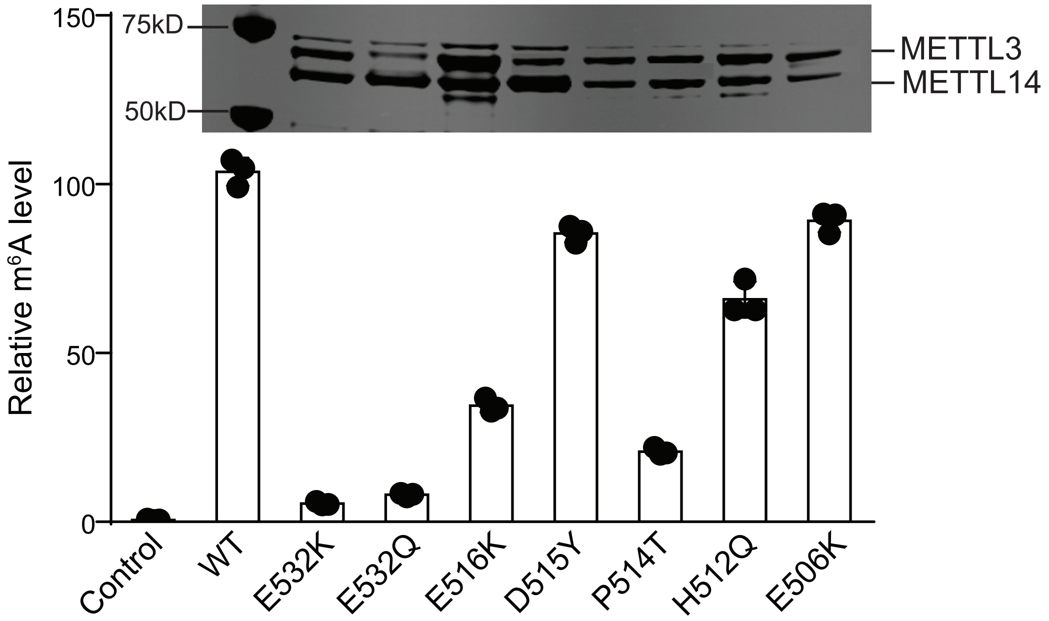

Supplementary Figure 6. METTL3 mutation affects its methylation activity in biochemistry assay.
Purified FLAG-tagged METTL14 with wildtype or mutant METTL3 was assessed by SDS-PAGE. The methyltransferase activity of the METTL3-METTL14 complex containing either METTL3 mutant or wild-type METTL3 was determined by measuring the ratio of *d3*-m6A to G by LC-MS/MS after incubation of the methyltransferase complex with a RNA probe, *n* = 4 (2 biological replicates and 2 technical replicates).

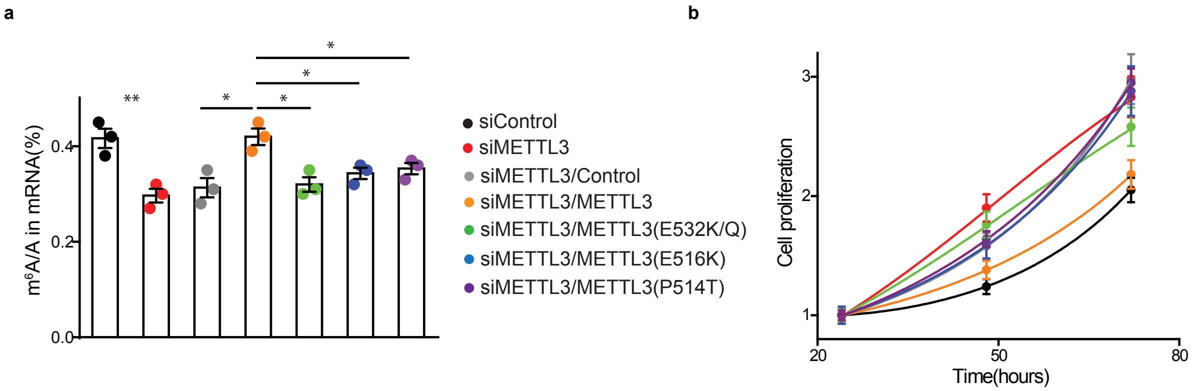

Supplementary Figure 7. Functional validation of METTL3 mutation in T24 bladder cancer cells

(a) Impaired m^{6}A RNA methyltransferase activity of mutant METTL3 in bladder cancer cell line "T24". LC-MS/MS quantification of the m^{6}A/A ratio in polyA-RNA in METTL3 or Control knockdown cells, rescued by overexpression of wildtype or mutant METTL3 is shown. **(b)** Mutant METTL3 decreased proliferation of "T24" cells. Proliferation of METTL3 or Control knockdown cells, rescued by overexpression of wildtype or mutant METTL3 in MTS assays is shown. Cell proliferation is calculated as the MTS signal at the tested time point normalized to the MTS signal ~ 24 hours after cell seeding. For all experiments in **(a-b)**, number of biological replicates is 3 and error bars indicate mean ± s.e.m. *, $p < 0.05$; **, $p < 0.01$ by t-test. Legend is shared between (a) and (b).

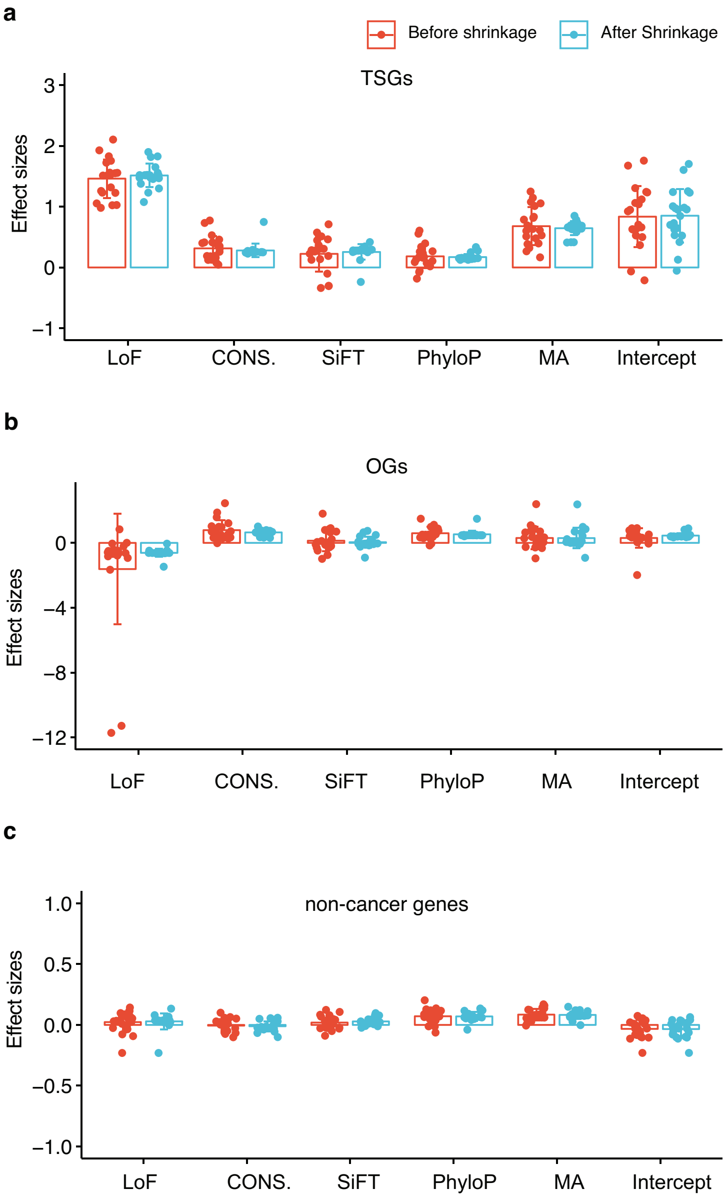

Supplementary Figure 8. Impact of ASH for functional feature effect size estimation
Effect sizes for five functional features and average increased mutation rate (the same as in Figure 2) before and after ASH. Each dot represents an estimate from one tumor type. Bars and error bars represent mean values and standard deviations across tumor types. **(a)** Estimation results for TSGs**. (b)** Estimation results for OGs**. (c)** Estimation results for the remaining genes. After ASH, values with large estimate standard errors were shrinked towards the mean. This avoided some extreme values as found in (b), estimates for LoF.

Tumor type	Parameter estimate (standard error)												
	$\beta_{0, t=1}$	$\beta_{0, t=2}$	$\beta_{0, t=3}$	$\beta_{0, t=4}$	$\beta_{0, t=5}$	$\beta_{0, t=6}$	$\beta_{0, t=7}$	$\beta_{0, t=8}$	$\beta_{0, t=9}$	β^{expr}	β^{repl}	β^{hic}	α
**BLCA**	-7.18(0.06)	-7.11(0.05)	-5.06(0.02)	-6.42(0.02)	-7.35(0.03)	-5.95(0.01)	-8.63(0.05)	-9.08(0.07)	-7.89(0.03)	-0.04(0.01)	0.03(0.01)	-0.05(0.01)	6.52(0.42)
**BRCA**	-7.79(0.09)	-8.09(0.09)	-5.8(0.03)	-7.99(0.04)	-8.06(0.04)	-7.34(0.02)	-9.06(0.07)	-9.08(0.07)	-8.42(0.04)	-0.09(0.02)	0.07(0.02)	-0.05(0.02)	4.8(0.66)
**CESC**	-9.37(0.19)	-8.86(0.12)	-6.29(0.03)	-8.14(0.05)	-8.92(0.06)	-7.63(0.02)	-10.83(0.17)	-11.02(0.19)	-10.14(0.09)	-0.04(0.02)	0.06(0.03)	-0.03(0.02)	4.3(0.84)
**CHOL**	-12.07(0.71)	-10.61(0.29)	-8.98(0.13)	-11.88(0.29)	-10.12(0.11)	-10.33(0.09)	-12.62(0.41)	-12.21(0.33)	-11.13(0.14)	0(0.07)	0.1(0.08)	-0.1(0.07)	8.91(NA)
**ESCA**	-8.88(0.14)	-7.55(0.06)	-5.95(0.03)	-8.88(0.06)	-7.68(0.03)	-7.8(0.02)	-9.41(0.08)	-9.12(0.07)	-8.56(0.04)	-0.07(0.02)	0.17(0.02)	-0.09(0.02)	3.5(0.42)
**GBM**	-9.68(0.2)	-9.04(0.13)	-5.68(0.03)	-9.75(0.09)	-9.34(0.07)	-8.44(0.03)	-10.02(0.1)	-10.39(0.12)	-9.01(0.05)	-0.05(0.02)	0.2(0.03)	-0.12(0.02)	1.26(0.1)
**LIHC**	-8.59(0.12)	-7.55(0.06)	-6.66(0.04)	-8.91(0.06)	-8.1(0.04)	-7.77(0.02)	-8.06(0.04)	-8.94(0.06)	-7.73(0.02)	-0.07(0.02)	0.13(0.02)	-0.06(0.02)	5.15(0.69)
**HNSC**	-7.41(0.07)	-6.84(0.04)	-5.07(0.02)	-7.3(0.03)	-7.32(0.03)	-6.64(0.01)	-8.27(0.04)	-9.08(0.07)	-7.92(0.03)	-0.05(0.01)	0.19(0.01)	-0.08(0.01)	4.5(0.29)
**KICH**	-12.22(0.71)	-10.94(0.32)	-7.96(0.07)	-11.46(0.22)	-10.51(0.13)	-10.02(0.07)	-11.36(0.21)	-11.17(0.19)	-9.62(0.07)	0(0.05)	0.16(0.06)	-0.05(0.05)	0.44(0.07)
**KIRC**	-8.84(0.13)	-8.32(0.09)	-6.99(0.04)	-9.14(0.07)	-8.4(0.05)	-8.14(0.03)	-8.81(0.06)	-9.28(0.07)	-8.43(0.04)	-0.01(0.02)	0.08(0.02)	0(0.02)	8.67(2.97)
**KIRP**	-8.88(0.14)	-8.35(0.09)	-7.53(0.06)	-9.21(0.07)	-8.82(0.06)	-8.42(0.03)	-9.02(0.07)	-9.68(0.09)	-8.55(0.04)	-0.07(0.02)	-0.04(0.03)	-0.02(0.02)	5.65(1.57)
**LUAD**	-6.54(0.04)	-5.21(0.02)	-5.28(0.02)	-6.88(0.02)	-5.71(0.01)	-6.3(0.01)	-6.91(0.02)	-8.31(0.04)	-7.37(0.02)	-0.1(0.01)	0.29(0.01)	-0.11(0.01)	2.14(0.06)
**LUSC**	-7.59(0.07)	-6.67(0.04)	-6.27(0.03)	-7.8(0.04)	-7.04(0.02)	-7.35(0.02)	-8.16(0.04)	-9.29(0.07)	-8.19(0.03)	-0.13(0.01)	0.25(0.02)	-0.1(0.02)	3.13(0.22)
**PAAD**	-11.51(0.5)	-10.18(0.22)	-6.95(0.04)	-11.12(0.19)	-10.55(0.13)	-9.66(0.06)	-11.07(0.17)	-10.85(0.16)	-10.6(0.1)	-0.09(0.04)	0.24(0.05)	-0.07(0.04)	1.43(0.31)
**PRAD**	-9.38(0.17)	-9(0.12)	-6.17(0.03)	-9.82(0.1)	-9.25(0.07)	-8.66(0.04)	-9.83(0.09)	-9.88(0.1)	-9.26(0.05)	-0.06(0.02)	0.22(0.03)	-0.05(0.02)	2.37(0.34)
**SARC**	-10.07(0.26)	-9.22(0.14)	-7.18(0.05)	-9.85(0.1)	-9.26(0.07)	-8.93(0.04)	-10.35(0.13)	-10.65(0.15)	-9.89(0.07)	-0.02(0.03)	0.19(0.04)	-0.13(0.03)	2.23(0.54)
**SKCM**	-8.08(0.09)	-7.9(0.07)	-4.28(0.01)	-9.07(0.07)	-8.13(0.04)	-4.88(0.01)	-8.45(0.05)	-8.6(0.05)	-7.7(0.02)	-0.05(0.01)	0.26(0.01)	-0.1(0.01)	1.7(0.04)
**TGCT**	-10.11(0.24)	-9.46(0.15)	-8.25(0.08)	-11.15(0.19)	-9.79(0.09)	-9.76(0.06)	-11.23(0.2)	-10.42(0.13)	-10.4(0.1)	0.12(0.05)	0.08(0.06)	-0.04(0.05)	0.35(0.05)
**UCEC**	-9.28(0.18)	-8.11(0.08)	-4.98(0.02)	-9.54(0.09)	-8.29(0.05)	-7.53(0.02)	-9.88(0.11)	-9.74(0.1)	-7.99(0.03)	-0.11(0.02)	0.03(0.02)	-0.05(0.02)	4.92(0.6)
**UCS**	-10.19(0.26)	-10.01(0.2)	-7.82(0.07)	-11.08(0.18)	-10.57(0.14)	-10.23(0.08)	-11.77(0.25)	-11.45(0.21)	-10.74(0.11)	-0.04(0.05)	0.17(0.06)	-0.13(0.05)	3.37(2.6)

Supplementary Table 1. Parameter estimation results for background mutation model (BMM).
For each tumor type, we obtained the maximum likelihood estimates for parameters and calculated standard errors derived from observed Fisher Information matrix. $\beta_{0t,}$ baseline mutation rate for type t, where $t=\{1,2,\ldots,9\}$ corresponds to 9 pre-defined mutation types (see Supplementary Notes for parameterization details). β^{expr} , β^{repl} and β^{hic} are effect sizes for background mutation features “expression”, “replication timing” and “HiC”, respectively. α is the hyperparameter for the gene specific mutation rate.

**Tumor type**	**χ^2 statistics**	***p* value**
**BRCA**	2308.4	0.00E+00
**SKCM**	2451.8	0.00E+00
**UCEC**	1669.6	0.00E+00
**BLCA**	1377.4	5.42E-297
**HNSC**	932.0	1.96E-200
**LUAD**	671.6	4.80E-144
**LIHC**	625.6	4.53E-134
**GBM**	488.8	1.74E-104
**CESC**	487.4	3.55E-104
**PRAD**	356.3	7.53E-76
**UCS**	271.2	1.78E-57
**LUSC**	267.5	1.11E-56
**KIRP**	174.7	1.05E-36
**ESCA**	109.9	7.58E-23
**KIRC**	57.8	8.34E-12
**SARC**	11.2	2.41E-02
**CHOL**	8.8	6.69E-02
**PAAD**	8.6	7.10E-02
**KICH**	-2.5	1.00E+00
**TGCT**	-70.3	1.00E+00

Supplementary Table 2. Model selection (with or without spatial effect) results for the OG model

We performed χ^2 difference test for model selection, the test statistics is given by $-2\times$ $(logP\left(Y^{NS}\middle|\hat{\beta}^b, \hat{\beta}^b_{0t}\, \hat{\alpha}, \hat{\beta}^f, \hat{\beta}^f_0\right) - logP\left(\mathrm{Y}^{\mathrm{NS}}\middle|\hat{\beta}^b, \hat{\beta}^b_{0t}\, \hat{\alpha}, \hat{\beta}^f, \hat{\beta}^f_0, \hat{p}_1, \hat{v}_{01}, \hat{v}_{10}, \hat{\rho}_0, \hat{\rho}_1\right))$ for OG data. The table is sorted by *p* values from low to high. See Supplementary Notes for details.

Tumor type	Novel cancer genes
**BLCA**	*ABHD15,ACTB,ADCY8,AGR3,AHR,ALDH16A1,C12orf43,C18orf8,C3orf70,C8orf76,CDKN1A,CLASP2,COX6A1,ELF3,EME1,EPS8,FOXQ1,FURIN,FZD7,GAR1,GNA13,HIST1H1E,HIST2H2BE,IKZF2,KCNF1,KCNK2,KHDRBS1,KHDRBS2,KIAA1522,KIR3DL1,KLF5,MEOX2,METTL3,MICALL1,MIF4GD,NAALADL1,NUP93,OGDH,PDSS2,PHF3,PHLDA3,PPCS,RAD51C,RALGPS1,RARS2,RERE,RFTN2,RHOB,RXRA,SF1,SF3A3,SHANK1,TAS2R9,TFPI2,TMCO4,TRAF3IP2,TTYH1,UNC93B1,XYLT2,ZBTB7B,ZFP36L1,ZNF750*
**BRCA**	*HSD3B2,MED23,MUC17,PCDHB7,RGS7,HIST1H2BC,DNASE1L3,WSCD2,SETDB1*
**CESC**	*ABCA12,C3orf70,HIST1H1B,HIST1H4K,MAPK1,MED1,SBNO1*
**CHOL**	*ATG16L1,DNAH5*
**ESCA**	*C10orf76,HDAC4,KPNB1,NAA16,PNLIPRP3,SLC39A12*
**GBM**	*IL18RAP,LZTR1,NUP210L,ODF4,SPINT1,TMEM147,TPTE2,UGT2A3,ZDHHC4*
**LIHC**	*CREB3L3,IRF2*
**HNSC**	*CSNK2A1,CUL3,FAT1,GPATCH8,HIST1H3C,MAPK1,NAA25,RASA1,SYT6,THSD7A,TP63,ZNF750*
**KIRC**	*COL11A1,CUL9,MOCOS*
**KIRP**	*CALCR,CUL3,FBXO47,KIAA0922,PARD6B,PCF11,SCRN2*
**LUAD**	*DZIP1L,LCE1F,MGA,PTPRU,SNRPD3,SOS1,ZFP36L1*
**LUSC**	*ADAMTS12,DPPA4,ELTD1*
**PRAD**	*CNTNAP1,COL11A1,CSMD3,CUL3,ETV3*
**SKCM**	*ARL16,DDX17,HTR3D,LCE1B,NPAS1,OXA1L,PCDHB8,PDE1A,REG4,RQCD1,SLC27A5,STK19,TACR3,TTC9*
**UCEC**	*COPB1,DEPDC1B,INPPL1,LYPLA2,LZTR1,METAP1,METTL14,MGA,MME,NAA30,OGDHL,RBM39,RRAS2,SIN3A,SLC30A9,SOS1,ST5,TFDP1,TTC38,ZNF485*
**UCS**	*SAMD4B,ZBTB7B*

Supplementary Table 3. List of novel cancer genes identified in each tumor type by driverMAPS.
Tumor types without any novel genes identified are not listed.

Position (on Chr14, hg19)	Mutation	log(Bayes Factor)	Amino acid change	Loss of function	Conser-vation	Sift_ pred	Phylop_ pred	MA_ pred	No. mutations
21967254	C>T	2.95	E516K	No	Yes	Yes	Yes	Yes	2
21971663	G>A	2.37	Q126X	Yes	Yes	Yes	Yes	No	1
21971651	C>A	2.17	E130X	Yes	Yes	No	Yes	No	1
21969218	T>A	1.48	N318I	No	Yes	Yes	Yes	Yes	1
21967257	C>A	1.48	D515Y	No	Yes	Yes	Yes	Yes	1
21967260	G>T	1.48	P514T	No	Yes	Yes	Yes	Yes	1
21967206	C>G	1.47	E532Q	No	Yes	Yes	Yes	Yes	1
21969223	G>C	1.47	F316L	No	Yes	Yes	Yes	Yes	1
21967206	C>T	1.47	E532K	No	Yes	Yes	Yes	Yes	1
21967452	C>T	1.47	E506K	No	Yes	Yes	Yes	Yes	1
21967728	C>T	1.47	E454K	No	Yes	Yes	Yes	Yes	1
21967676	C>T	1.45	R471H	No	Yes	Yes	Yes	Yes	1
21967685	C>T	1.45	R468Q	No	Yes	Yes	Yes	Yes	1
21971844	G>A	0.91	P94L	No	Yes	Yes	Yes	No	1
21969985	C>T	0.69	E262K	No	Yes	No	Yes	No	1
21967264	A>C	0.59	H512Q	No	Yes	No	No	No	1
21972010	C>T	0.40	E39K	No	No	No	Yes	No	1

Supplementary Table 4. Nonsysnonymous mutations in METTL3 in BLCA cohort.
For each mutation, we calculated a Bayes factor only using data at the mutated position, which is the likelihood of H_{TSG} versus H_0 (since METTL3 was identified as a TSG in BLCA cohort) at the mutated position. Mutations in this table were ordered based on log(Bayes factor) from high to low, prioritizing mutations contributing more towards the gene being a TSG. But one thing to note is that the gene-level log Bayes factor calculated by driverMAPS is not merely an add up of log Bayes factor for all individual positions in the gene, this is because the positions are not independent and share gene-level random effect.

Supplementary Note 1: Data preparation

Lists of somatic mutations from cancer sequencing studies

TCGA GDAC Firehose (https://gdac.broadinstitute.org/) (version: analyses___2016_01_28). We obtained the MAF (Mutation Annotation Format, .maf) files using firehose_get (version 0.4.6) (https://confluence.broadinstitute.org/display/GDAC/Download). When several versions of the MAF file were obtained for one tumor type (which can occur when the original MAF file has been filtered against several mutation blacklist files) then we took the intersection among MAF files (i.e., we only used mutations that were retained in all MAF files.) We only used MAF files that were originally aligned to genome build hg19 (GRCh37 Genome Reference Consortium Human Reference 37 (GCA_000001405.1)). This left us with 27 tumor types. We excluded THYM (Thymoma) and PCPG (Pheochromocytoma and Paraganglioma) as there were insufficient mutations (< 2000). We ran driverMAPS on the remaining 25 tumor types, extracting position and nucleotide change information for all single-nucleotide somatic mutations from the MAF files. After inspecting the results, we excluded results from five tumor types – ACC (Adrenocortical carcinoma), LGG (Brain Lower Grade Glioma), THCA (Thyroid carcinoma), and DLBC (Lymphoid Neoplasm Diffuse Large B-cell Lymphoma), and STAD (Stomach adenocarcinoma) – before doing comparisons with other software and other downstream analyses. The first four of these tumor types all had 10 or more novel driver genes identified, and the vast majority (>90%) of these were caused by recurrent mutations that appeared to be false positives based on visual inspection of read alignment plots. STAD has 111 significant genes identified and contained >1000 mutations within these genes, making it burdensome to visually evaluate whether the mutations are false positives. The 20 remaining tumor types, and their abbreviations, used in the paper are as follows:

- Breast invasive carcinoma, BRCA
- Cervical squamous cell carcinoma and endocervical adenocarcinoma, CESC
- Cholangiocarcinoma, CHOL
- Esophageal carcinoma, ESCA
- Glioblastoma multiforme, GBM
- Head and neck squamous cell carcinoma, HNSC
- Kidney chromophobe, KICH
- Kidney renal clear cell carcinoma, KIRC
- Kidney renal papillary cell carcinoma, KIRP
- Liver hepatocellular carcinoma, LIHC
- Lung adenocarcinoma, LUAD
- Lung squamous cell carcinoma, LUSC
- Pancreatic adenocarcinoma, PAAD
- Prostate adenocarcinoma, PRAD
- Sarcoma, SARC
- Skin cutaneous melanoma, SKCM
- Testicular germ cell tumors, TGCT
- Uterine carcinosarcoma, UCS
- Uterine corpus endometrial carcinoma, UCEC

Filtering of mutations

The lists of somatic mutations obtained above have a substantial number of false positive mutations. Because our method is designed to be very sensitive to capture mutation patterns that deviate from the background mutation process it is important to perform QC-filtering to attempt to remove false positives.

1. We filtered out mutations with less than 4 reads supporting the alternate allele or with less than 5% alternate allele frequency.

2. We filtered out mutations that are directly adjacent to one another and identified from the same tumor-normal pair. Such mutations are mostly di-nucleotide or multi-nucleotide mutations instead of consecutive single nucleotide mutations.
3. We filtered out mutations commonly seen as single-nucleotide polymorphisms (SNPs), defined as having minor allele frequency exceeding 5% in any ancestry group of the ExAc, 1000G and ESP6500 databases (see ANNOVAR[1] documentation, database made date: 20150413).
4. We excluded hyper-mutated tumor samples, following the procedure of[2]. Specifically we excluded samples with more than (Q3 + IQR*4.5) mutations, where Q3 denotes the third quartile of mutation counts across the corresponding tumor type, and IQR denotes the interquartile range.
5. When multiple tumor specimens were sequenced with the same matching normal tissue we picked one of the tumor specimens at random and only include somatic mutations from this tumor specimen. We removed the small number of samples that had multiple samples from normal tissue.
6. We used the additional pre-processing procedures provided by the MutSig software suite to filter out known sequencing artifacts.

The filtered lists of mutations for all 20 tumor types are available at https://szhao06.bitbucket.io/driverMAPS-documentation/docs/download.html.

Generating mutation count files

For each of the 20 tumor types, we generated a mutation count file. This file lists all possible mutations that could occur at each sequenced position.

To obtain the sequenced positions for each tumor cohort, we extracted the exonic DNA capture kit information for all samples in each cohort from Genomic Data Commons (GDC, https://portal.gdc.cancer.gov/legacy-archive/search/f). The regions targeted by each kit (30Mb to ~60Mb depending on the capture array) were obtained from the manufacturer's website, and regions that were targeted in all samples of a cohort were retained. We then excluded mapping blacklisted regions using two blacklist files,

- wgEncodeDacMapabilityConsensusExcludable.bed.gz and
- wgEncodeDukeMapabilityRegionsExcludable.bed.gz

obtained from http://hgdownload.cse.ucsc.edu/goldenpath/hg19/encodeDCC/wgEncodeMapability/.

From these sequenced positions we generated a list of possible mutations. Each sequenced position has three nucleotides that it could possibly mutate to, so the total number of possible mutations is three times the number of sequenced positions. For each possible mutation we counted how many were observed in the MAF file, aggregating across all individuals. Depending on the mutation rate and cohort size, we observed ~200 to ~4500 mutations per Mb, so the mutation counts for the vast majority of possible mutations were 0.

Supplementary Note 2: Genomic annotation

We used ANNOVAR[1] to add annotations to all possible mutations (including those with 0 count) in the mutation count file described above.

Gene and mutation type information

We added gene information based on the GENCODE database (https://www.gencodegenes.org/, version 19, Feb 2014). We annotated each mutation as being in one of the following categories (order indicates precedence): splicing (+/- exon-intron boundary), exonic-stopgain, exonic-stoploss, exonic-nonsynonymous SNV, exonic-synonymous SNV, ncRNA, upstream, UTR3, UTR5, intronic, downstream.

Background features

Our background mutation model includes several annotations that may affect background mutation rates.

To account for the different mutation rates that may occur in different sequence contexts we annotated each possible mutation as being one of 9 possible types:

1. C:G to G:C mutations at CpG dinucleotides
2. C:G to A:T mutations at CpG dinucleotides
3. C:G to T:A mutations at CpG dinucleotides
4. C:G to G:C mutations not in CpG dinucleotides
5. C:G to A:T mutations not in CpG dinucleotides
6. C:G to T:A mutations not in CpG dinucleotides
7. A:T to T:A mutations
8. A:T to C:G mutations
9. A:T to G:C mutations

We also annotated each mutation using three gene-level features: gene expression, replication timing and chromatin conformation measured by HiC sequencing. These same features are used in running MutsigCV. Data for these three features were downloaded from http://archive.broadinstitute.org/cancer/cga/mutsig.

Functional annotations

We considered several mutation-level functional annotations. Including more features will improve fit of the model but also at the risk of overfitting. As many scores are highly correlated, we eventually selected 5 annotations that assess mutation functional impact in complementary ways:

- The "Loss of function (LoF)" annotation indicates if the mutation is either a nonsense or splice site mutation.
- The "Conservation" feature is an indicator based on multiple alignment of amino acid sequence (http://hgdownload.soe.ucsc.edu/goldenPath/hg19/multiz100way/). We annotated the position as "conserved" if it encodes an amino acid that is highly conserved in 100-way multiple alignment (less than 3 species showed distinct amino acid at this position).
- The remaining three features are phyloP[3], SiFT[4] and MutationAssessor[5] predictions, all obtained from ANNOVAR.

Supplementary Note 3: Statistical model description

Note: this section is partially redundant with Methods, but adds additional details.

Since we model each tumor type cohort separately we describe the model for a single cohort. Let Y_{it} denote the total number of mutations (across all samples in the cohort) of type t at sequence position i, where $t \in 1, .., 9$ refers to the 9 nucleotide change types described earlier. Let NS denote the set of pairs (i, t) such that a mutation of type t at sequence position i would be non-synonymous. Similarly, let S denote the set of pairs (i, t) representing synonymous mutations.

In practice, we treated synonymous mutations with high splicing impact score (dbscSNV version 1.1 for splice site prediction by AdaBoost and Random Forest[6] and spidex[7]) as nonsynonymous mutations since they are likely to be functional; i.e. they were included in NS instead of S. We refer to mutations in S as S mutations and mutations in NS as NS mutations.

We let S_g denotes the subset of all S mutations in gene g, and Y^{S_g} denote the corresponding observed counts.

$$Y^{S_g} = \{Y_{it} : (i, t) \in S_g\}. \tag{1}$$

We use analogous notation, NS_g, Y^{NS_g} for NS mutations.

Background Mutation Model (BMM)

For S mutations we assume the following "background mutation model":

$$Y_{it}|H_m \sim \text{Poisson}(\mu_{it}\lambda_{g(i)})[\text{for}(i,t) \in S] \tag{2}$$

where μ_{it} represents a background mutation rate (BMR) for mutation type t at position i, and $\lambda_{g(i)}$ represents a gene-specific effect for the gene $g(i)$ that contains sequence position i. Note that the parameters of this BMM do not depend on the model m, so $P(Y^{S_g}|H_m)$ is the same for all m.

Because at a position i only certain mutation types are possible, in equation (2), values of t are limited to mutation types (t) that are possible at i. This could be expressed more formally by defining an indicator, δ_{it}, which takes the value 1 if mutation of type t is possible at i and 0 otherwise, and writing equation (2) as $Y_{it} \sim \delta_{it}\text{Poisson}(\mu_{it}\lambda_g(i))$. For simplicity, we make this indicator implicit, and so equations below apply only to t such that $\delta_{it} = 1$.

We allow the BMRs to depend on background features (e.g. the expression level of the gene) using a log-linear model:

$$\log \mu_{it} = \beta^b_{0t} + \sum_j x^b_{ij}\beta^b_j, \tag{3}$$

where x^b_{ij} denotes the j-th background feature of position i (not dependent on mutation type), β^b_{0t} controls the baseline mutation rate of type t, and β^b_j is the coefficient of the j-th feature. The values x^b_{ij} are observed, and the parameters β^b are to be estimated. To indicate the dependence of μ_{it} on parameters β^b we write $\mu_{it}(\beta^b)$.

We assume that the gene-specific effects λ_g have a gamma distribution across genes:

$$\lambda_g \sim \text{Gamma}(\alpha, \alpha), \tag{4}$$

where α is a hyperparameter to be estimated.

Selection Mutation Model (SMM)

For non-synonymous mutations we introduce additional model-specific parameter: γ^m_{it}, which represent a selection effect (SE) for mutation type t at position i under model m; and θ^m_i, which represents a spatial effect for position i under model m:

$$Y_{it}|H_m \sim \text{Poisson}(\mu_{it}\lambda_{g(i)}\gamma^m_{it}\theta^m_i) \text{ [for } (i,t) \in NS]. \tag{5}$$

For all models, we allow the selection effect, γ^m_{it}, to depend on functional features (e.g. the assessed deleteriousness of the mutation), using a log-linear model:

$$\log \gamma^m_{it} = \beta^{f,m}_0 + \sum_j x^f_{ijt}\beta^{f,m}_j, \tag{6}$$

where x^f_{ijt} denotes the j-th functional feature of position i (this depends on mutation type; e.g. at the same position, some mutations may be more deleterious than others), $\beta^{f,m}_j$ is the coefficient of the j-th functional feature and the intercept $\beta^{f,m}_0$ captures the overall change of mutation rate at NS sites regardless of functional impact. To indicate the dependence of γ^m_{it} on parameters $\beta^{f,m}$ we write $\gamma_{it}(\beta^{f,m})$.

The "spatial effect" θ^m_i, accounts for spatial clustering of mutations (including recurrent mutations at the same position in different samples). We assume that at each position i, either $\theta^m_i = \rho^m_1 \geq 1$ (if position i is

in a mutation hotspot) or $\theta_i^m = \rho_0^m \leq 1$ (if position i is not in a mutation hotspot). Thus the ratio ρ_1^m/ρ_0^m represents the average increase of mutation rate in hotspots vs outside hotspots under model m. We use a Hidden Markov Model(HMM) to model hotspot status. Specifically, we let Z_i^m be an indicator of whether position i is in a hotspot ($Z_i^m = 1$) or not ($Z_i^m = 0$), and assume Z_i^m follows a Markov chain with:

- Initial state probabilities: $p_0^m = P(Z_1^m = 0)$, $p_1^m = P(Z_1^m = 1)$, where $p_0^m + p_1^m = 1$
- Transition matrix:

$$\begin{array}{ccc} & Z_{i+1}^m = 0 & Z_{i+1}^m = 1 \\ Z_i^m = 0 & v_{00}^m & v_{01}^m \\ Z_i^m = 1 & v_{10}^m & v_{11}^m \end{array}$$

We use Θ^m to denote the set of all parameters related to this HMM for model m: $\Theta^m = \{p_0^m, p_1^m, v_{00}^m, v_{01}^m, v_{10}^m, v_{11}^m, \rho_0^m, \rho_1^m\}$.

Supplementary Note 4: Parameter estimation

Note: this "parameter estimation" section is partially redundant with Methods, but adds additional details.

Estimating parameters in BMM

We first infer parameters related to the background mutational model: $\boldsymbol{\beta}^b$ (the vector of coefficients and the intercepts) and α. We use only S mutations in this step. Recall that S_g denotes the subset of S mutations in gene g, and Y^{S_g} denotes the corresponding observed counts. The likelihood for gene g is then given by:

$$\begin{aligned} P(Y^{S_g}|\boldsymbol{\beta}^b, \alpha) &= \int \prod_{i,t \in S_g} P(Y_{it}|\mu_{it}(\beta^b), \lambda_g) p(\lambda_g|\alpha) d\lambda_g \\ &= \int \prod_{i,t \in S_g} \frac{\mu_{it} y_{it}}{y_{it}!} \frac{\Gamma(\alpha + y_+^{S_g})}{\Gamma(\alpha)} \frac{\alpha^\alpha}{(\alpha + \mu^{S_g})^{\alpha + y_+^{S_g}}}, \end{aligned} \tag{7}$$

where $y_+^{S_g}$ denotes the observed number of S mutations in gene g, and μ^{S_g} denotes the expected number of S mutations in gene g . (Note that the product here is over *all* i, t that are S mutations, including those that have observed count 0 ($Y_{it} = 0$), because these form part of the data and so contribute to the likelihood.)

We assume independence across genes to obtain a likelihood for synonymous mutations:

$$L^S(\beta^b, \alpha) := \prod_g P(Y^{S_g}|\beta^b, \alpha). \tag{8}$$

We use numerical methods (the BFGS algorithm implemented in the R function optim) to maximize this likelihood and obtain maximum likelihood estimates $\hat{\boldsymbol{\beta}}^b, \hat{\alpha}$,

Estimating parameters in SMM

We next estimate the model-specific parameters $\boldsymbol{\beta^{f,m}}$ for all models. During this step we ignore the HMM model (i.e. we set $\theta_i^m = 1$), motivated by the fact that spatially-clustered mutations are relatively rare and so should not significantly impact the estimates of $\boldsymbol{\beta^{f,m}}$

For $m = OG, TSG$ we estimate $\boldsymbol{\beta^{f,m}}$ using the NS mutation data from two different curated lists: G_{OG} containing 53 OGs (used for $m = OG$) and G_{TSG} containing 71 TSGs (used for $m = TSG$). For the null model, we used the remaining genes excluding these 53 OGs and 71 TSGs as the training set, as the vast majority of the remaining genes should not be driver genes (our parameter estimation results showed in

Figure 2c, last panel shows all $\boldsymbol{\beta^f}$ for $m = H_0$ are close to 0 are consistent with this claim). Let G_m denote these sets of training genes.

Assuming independence across genes, the likelihood for $\boldsymbol{\beta^{f,m}}$ is:

$$L(\beta^{f,m}) = \prod_{g\in G_m} P(Y^{NS_g}, Y^{S_g}|\beta^{f,m}) \propto \prod_{g\in G_m} P(Y^{NS_g}|\beta^{f,m}, Y^{S_g}). \tag{9}$$

The term in this likelihood for gene g is given by:

$$P(Y^{NS_g}|\beta^{f,m}, Y^{S_g}) = \int \prod_{i,t\in NS_g} P(Y_{it}|\mu_{it}(\hat{\beta^b}), \gamma_{it}(\beta^{f,m}), \lambda_g) P(\lambda_g|Y^{S_g}, \hat{\alpha}) d\lambda_g.$$

$$= \prod_{i,t\in NS_g} \frac{(\mu_{it}(\hat{\boldsymbol{\beta}}^b)\gamma_{it}(\boldsymbol{\beta^{f,m}}))^{y_{it}}}{y_{it}!} \frac{(\hat{\alpha} + \mu^{S_g}(\hat{\boldsymbol{\beta}}^b))^{\hat{\alpha}+y_+^{S_g}}}{\Gamma(\hat{\alpha} + y_+^{S_g})} \frac{\Gamma(\hat{\alpha} + y_+^{S_g} + y_+^{NS_g})}{(\hat{\alpha} + \mu^{S_g}(\hat{\boldsymbol{\beta}}^b) + \mu^{NS_g})^{\hat{\alpha}+y_+^{S_g}+y_+^{NS_g}}}, \tag{10}$$

where $y_+^{NS_g}$ denotes the total number of observed number of NS mutations in gene g and μ^{NS_g} denotes the expected number of NS mutations in gene g, which is a function of $\boldsymbol{\beta^{f,m}}$. This expression comes from the fact that

$$\lambda_g|Y^{S_g}, \hat{\alpha} \sim \text{Gamma}(\hat{\alpha} + y_+^{S_g}, \hat{\alpha} + \mu^{S_g}(\hat{\boldsymbol{\beta}}^b)), \tag{11}$$

where $\mu^{S_g}(\hat{\boldsymbol{\beta}}^b)$ and $y_+^{S_g}$ are, respectively, the expected (considering only mutational features) and observed number of synonymous mutations in gene g. (Note that the conditional mean of this distribution is $\frac{\hat{\alpha}+y_+^{S_g}}{\hat{\alpha}+\mu^{S_g}(\hat{\boldsymbol{\beta}}^b)}$, so if $y_+^{S_g} > \mu^{S_g}(\hat{\boldsymbol{\beta}}^b)$, then $E(\lambda_g|Y^{S_g}, \hat{\alpha}) > 1$.)

We used numerical methods (the BFGS algorithm implemented in the R package optim) to maximize the likelihood (equation (10)) and obtain maximum likelihood estimates $\beta^{f,m}$. We also computed the standard errors of our estimates using the standard asymptotic distribution of the MLE. In tumor types with low mutation rates, or with low sample sizes, the standard errors of the selection parameters can be relatively large, so we borrowed information from other tumor types to "stabilize" these estimates. We implemented this using Adaptive Shrinkage (ASH)[8]. The intuition is that, we can estimate a population mean of $\boldsymbol{\beta}^{f,m}$ across all tumor types, and "shrink" the estimated value of $\boldsymbol{\beta}^{f,m}$ towards the population mean. This shrinkage effect is larger for tumor types with larger standard errors. We take the mean of the shrunken parameter estimates as the final estimates for $\boldsymbol{\beta}^{f,m}, \beta_0^{f,m}$, so all tumor types share the same parameter values in modeling γ_{it}.

HMM parameters

After estimating $\boldsymbol{\beta}_{f,m}$, we fix their values and estimate the HMM parameters Θ^m by maximum likelihood. We used the maximum likelihood estimation routines for HMMs implemented in the R package depmixS4[9], after editing the code to account for our emission probabilities which incorporate the gene-specific random effects. Specifically the emission probabilities are given by:

$$P(Y_i|Z_i) = \prod_{t:(i,t)\in NS_g} P(Y_{it}|Z_i) \tag{12}$$

$$Y_{it}|Z_i \sim Poisson(\mu_{it}(\hat{\boldsymbol{\beta}^b})\hat{\lambda}_{g(i)}\gamma_{it}(\hat{\beta^{f,m}})\rho^m_{Z_i}) \text{ [for}(i,t) \in NS_g\text{, where } g \in G_m] \tag{13}$$

Here, $\hat{\lambda}_{g(i)}$ denotes the posterior mean of $\lambda_{g(i)}(\hat{\alpha})$.

We estimated the HMM parameters for each tumor type separately. Because some tumor types have relatively few mutations and few mutation hotspots, we performed model selection to evaluate whether to use the HMM model for spatial effect. Specifically we computed the likelihood ratio for the model with vs without spatial

effect and performed a Chi-squared test. For $m = TSG$ and the null model, we found little evidence for clustering and so we ignored the HMM part (i.e. equivalently to setting $\rho_0^{TSG} = \rho_1^{TSG} = 1$ and $\rho_0^0 = \rho_1^0 = 1$). For $m = OG$ we found 5 tumor types with $p > 0.001$ and we ignored the HMM in the same way. The remaining 15 tumor types showed strong spatial effect (p $< 10^{-11}$).

In reality, hotpots in TSGs and non-cancer genes may be rare but still exist. Given more data in the future one could possibly capture such effects.

Supplementary Note 5: Gene classification

Having estimated the model parameters as above, for each gene g, we compute its Bayes Factor(BF) for being a driver gene as:

$$BF_g := \frac{0.5P(Y^{NS_g}, Y^{S_g}|H_{OG}) + 0.5P(Y^{NS_g}, Y^{S_g}|H_{TSG})}{P(Y^{NS_g}, Y^{S_g}|H_0)}. \tag{14}$$

The equal weights in the numerator of this BF assume that OGs and TSGs are equally common.

This BF simplifies to

$$BF_g = \frac{0.5P(Y^{NS_g}|Y^{S_g}, H_{OG}) + 0.5P(Y^{NS_g}|Y^{S_g}, H_{TSG})}{P(Y^{NS_g}|Y^{S_g}, H_0)}. \tag{15}$$

because $P(Y^{S_g}|H_m)$ is the same for every m. Computing the terms $P(Y^{NS_g}|Y^{S_g}, H_m)$ is performed using equation (10) above, substituting the estimated model parameters for each model m. After obtaining the BFs, we can compute the posterior probability of being a driver gene ($m = TSG, OG$) for every gene.

Let $\pi = 1 - P(H_0)$. Parameter π is the prior of a gene being a driver gene (either as TSG or OG) or the proportion of driver genes. The posterior of gene g being a driver gene:

$$P(H_m, m \neq 0|Y_g) = P(H_m, m \neq 0)P(Y_g|H_m, m \neq 0)/P(Y_g) = \frac{\pi BF_g}{\pi BF_g + 1 - \pi}. \tag{16}$$

The likelihood given π is:

$$P(Y|\pi) = \prod_g [\pi P(Y_g|H_m, m \neq 0) + (1-\pi)P(Y_g|H_0)] \propto \prod_g (\pi BF_g + 1 - \pi). \tag{17}$$

We obtained the MLE for π using an EM algorithm. Let η_g be the indicator of gene g being a cancer driver. The joint probability of Y_g, η_g is given by:

$$P(Y_g, \eta_g|\pi) \propto \pi^{\eta_g}(1-\pi)^{1-\eta_g} BF_g^{\eta_g}. \tag{18}$$

The Q function (expected complete data log-likelikhood) in the EM algorithm is given by:

$$Q(\pi|\pi^{(t)}) = \sum_g E_{\eta_g|Y_g,\pi^{(t)}} log(P(Y_g, \eta_g|\pi)). \tag{19}$$

Let h_g denote the probability for gene g being a driver gene. At the E step, we calculate h_g as:

$$h_g^{(t)} = \frac{\pi^{(t)} BF_g}{\pi^{(t)} BF_g + 1 - \pi^{(t)}}. \tag{20}$$

Now the Q function can be expressed as

$$Q(\pi|\pi^{(t)}) = \sum_g \sum_{\eta_g} P(\eta_g|\pi^{(t)}, Y_g) logP(Y_g, \eta_g|\pi) \tag{21}$$

$$= \sum_g [h_g^{(t)}(\log\pi + \log(BF_g)) + (1 - h_g^{(t)})log(1-\pi)]. \tag{22}$$

So at M step, we update π as

$$\pi^{t+1} = \frac{\sum_g h_g^{(t)}}{N}, \tag{23}$$

where N is the number of genes.

After estimating π we compute the posterior probability for each gene being a driver gene. We perform multiple testing correction to control FDR by a direct posterior probability approach[10]. We used FDR < 0.1 for calling significant driver genes.

Supplementary Note 6: Comparison with other software

Running other software

The same set of mutation list files (https://szhao06.bitbucket.io/driverMAPS-documentation/docs/download.html) used for driverMAPS were processed into input files, which were used to run each method in-house. **MutSigCV**[11] identifies driver genes as those with an excess number of mutations from the background mutational process. MutSigCV (v1.4) was run in MATLAB with the default parameters.

OncodriveFM[12]identifies driver genes as those possessing a bias towards accumulating variants with high functional impact. Silent mutations were assigned the least-damaging scores for each metric (0, 1, and -5.545, respectively), while inactivating mutations (primarily nonsense and splice site) were assigned the most-damaging scores (1, 0, and 5.975, respectively). OncodriveFM version 1.0.3 was run in Python 3 with the default parameters.

OncodriveFML[13] assesses the functional impact of tumor somatic mutations by running a simulation of relevant mutational processes, allowing it to directly compute FM bias and thus identify driver genes. OncodriveFML version 2.0.2 was run in Python 3 with the default parameters for testing coding regions, using the default precomputed CADD scores downloaded via the tool and the coding DNA sequence (CDS) regions file downloaded from the website.

OncodriveCLUST[14] identifies driver genes as those with a bias towards spatial clusters of mutations, comparing nonsynonymous mutations against a baseline model constructed from silent mutations. OncodriveCLUST version 1.0.0 was run in Python 3 using the gene transcripts file downloaded from the website and default parameters, except the minimum mutations threshold was lowered to 1 (default is 5).

dNdScv[15] was run as a R package (version: dndscv_0.0.0.9). For substitution models, we chose the option '192r_3w'. All other parameters were under default settings.

CBaSE[16] CBaSE v1.1 standalone version was run as suggested by the software website.

Comparison of precision

We defined a set of known cancer genes as the union of COSMIC cancer census genes (version 76)[17], gene list from[2] and[18]. These ended up with 713 "known" genes. We defined precision as the percentage of "known"

genes out of all significant genes. For all methods tested, we used FDR=0.1 as cut off for significant genes. For all unknown genes with FDR <0.1 from each methods (except for oncodriveFM and oncodriveCLUST, which called hundreds or thousands of unknown significant genes), we examined reads alignment (bam files were obtained from Genomic Data Commons) at called mutated positions by visual inspection to ensure the mutations are real. Genes with >20% false mutations were filtered and we provide the blacklist file used in this filtering step on the software website. As driverMAPS used a subset of known cancer genes (n=124) as the training set to get model parameters, to avoid the bias towards this subset of genes when calculating precision for driverMAPS, we implemented a leave-one-out strategy. Specifically, in each run, when we train the model, we leave one TSG/OG out. So data from this TSG/OG was never used at the training step. Then we get significant genes using the model trained in this run. We repeated this for every TSG/OG. Then, when calculating precision for driverMAPS, a TSG/OG is only considered significant if it is significant in the run without using it in the training step. All data related to driverMAPS (basic, +feature and full version) we presented in Figure 4 was obtained in this way. We found the model parameters are stable in each run and the overall result is very similar to the run not using this "leave-one-out" strategy. We reasoned that this benefits from the fact that driverMAPS estimates parameters from 20 tumor types and used ASH to shrink parameters towards the mean to avoid large deviations.

Comparison of power

We used the number of significant genes at FDR<0.1 for power comparison. Similarly, we again used the leave-one-out strategy and the TSG/OG used in the training set is only considered significant if it is significant in the run without using it in the training step.

Supplementary Note 7: Additional information for the paper

Primers

METTL3E532KFor ggcactcgcaagattAagttatttggacgacca

METTL3E532KRev tggtcgtccaaataactTaatcttgcgagtgcc

METTL3E532QFor ggcactcgcaagattCagttatttggacgacca

METTL3E532QRev tggtcgtccaaataactGaatcttgcgagtgcc

METTL3E516KFor agtcataaaccagatAaaatctatggcatgatt

METTL3E516KRer aatcatgccatagatttTatctggtttatgact

METTL3D515YFor accagtcataaaccaTatgaaatctatggcatg

METTL3D515YRev catgccatagatttcatAtggtttatgactggt

METTL3P514TFor tccaccagtcataaaAcagatgaaatctatggc

METTL3P514Trev gccatagatttcatctgTtttatgactggtgga

METTL3H512QFor gttcgttccaccagtcaGaaaccagatgaaatc

METTL3H512QRev gatttcatctggtttCtgactggtggaacgaac

METTL3E506KFor gatgtgatcgtagctAaggttcgttccaccagt

METTL3E506KRev actggtggaacgaacctTagctacgatcacatc

METTL3E454KFor tatgaacgggtagatAaaattatttgggtgaag

METTL3E454KRev cttcacccaaataatttTatctacccgttcata

Supplementary References

1. Wang K, Li M, Hakonarson H. ANNOVAR: Functional annotation of genetic variants from high-throughput sequencing data. *Nucleic acids research.* 2010;38(16):e164–e164.

2. Kandoth C, McLellan MD, Vandin F, et al. Mutational landscape and significance across 12 major cancer types. *Nature.* 2013;502(7471):333.

3. Pollard KS, Hubisz MJ, Rosenbloom KR, Siepel A. Detection of nonneutral substitution rates on mammalian phylogenies. *Genome research.* 2010;20(1):110–121.

4. Ng PC, Henikoff S. SIFT: predicting amino acid changes that affect protein function. *Nucleic Acids Research.* 2003;31(13):3812–3814.

5. Reva B, Antipin Y, Sander C. Predicting the functional impact of protein mutations: application to cancer genomics. *Nucleic acids research.* 2011;39(17):e118–e118.

6. Jian X, Boerwinkle E, Liu X. In silico prediction of splice-altering single nucleotide variants in the human genome. *Nucleic acids research.* 2014;42(22):13534–13544.

7. Xiong HY, Alipanahi B, Lee LJ, et al. The human splicing code reveals new insights into the genetic determinants of disease. *Science.* 2015;347(6218):1254806.

8. Stephens M. False discovery rates: a new deal. *Biostatistics.* 2017;18(2):275–294.

9. Visser I, Speekenbrink M, others. DepmixS4: An r package for hidden markov models.

10. Newton MA, Noueiry A, Sarkar D, Ahlquist P. Detecting differential gene expression with a semiparametric hierarchical mixture method. *Biostatistics.* 2004;5(2):155–176.

11. Lawrence MS, Stojanov P, Polak P, et al. Mutational heterogeneity in cancer and the search for new cancer-associated genes. *Nature.* 2013;499(7457):214.

12. Gonzalez-Perez A, Lopez-Bigas N. Functional impact bias reveals cancer drivers. *Nucleic acids research.* 2012;40(21):e169–e169.

13. Mularoni L, Sabarinathan R, Deu-Pons J, Gonzalez-Perez A, López-Bigas N. OncodriveFML: a general framework to identify coding and non-coding regions with cancer driver mutations. *Genome Biology.* 2016;17(1):128.

14. Tamborero D, Gonzalez-Perez A, Lopez-Bigas N. OncodriveCLUST: exploiting the positional clustering of somatic mutations to identify cancer genes. *Bioinformatics.* 2013;29(18):2238–2244.

15. Martincorena I, Raine KM, Gerstung M, et al. Universal Patterns of Selection in Cancer and Somatic Tissues. *Cell.* 2017;171(5):1029–1041.e21.

16. Weghorn D, Sunyaev S. Bayesian inference of negative and positive selection in human cancers. *Nature Genetics.* 2017;49(12):1785–1788.

17. Forbes SA, Beare D, Boutselakis H, et al. COSMIC: somatic cancer genetics at high-resolution. *Nucleic Acids Research.* 2017;45(D1):D777–D783.

18. Vogelstein B, Papadopoulos N, Velculescu VE, et al. Cancer Genome Landscapes. *Science.* 2013;339(6127):1546 LP–1558.
