# Supplementary material for: Detailed modeling of positive selection improves detection of cancer driver genes
Source: Nat Commun. 2019 Jul 30;10:3399. doi: 10.1038/s41467-019-11284-9 (PMC6667447; doi:10.1038/s41467-019-11284-9)
Supplement: Supplementary file 3 — Description of Additional Supplementary Files [file 41467_2019_11284_MOESM3_ESM.pdf]

Description of Additional Supplementary Files

File Name: Supplementary Data 1
Description: Parameter estimation results for modeling spatial effect (HMM) for OGs

File Name: Supplementary Data 2
Description: Gene function for novel significant genes from breast, lung and prostate cancers

File Name: Supplementary Data 3
Description: Genes found by loss-of-function screening affecting cell growth
