# Supplementary material for: Detailed modeling of positive selection improves detection of cancer driver genes
Source: Nat Commun. 2019 Jul 30;10:3399. doi: 10.1038/s41467-019-11284-9 (PMC6667447; doi:10.1038/s41467-019-11284-9)
Supplement: Supplementary file 7 — Reporting Summary [file 41467_2019_11284_MOESM7_ESM.pdf]

nature research

Corresponding author(s): Matthew Stephens and Xin He

Reporting Summary

Nature Research wishes to improve the reproducibility of the work that we publish. This form provides structure for consistency and transparency in reporting. For further information on Nature Research policies, see Authors & Referees and the Editorial Policy Checklist.

Statistical parameters

When statistical analyses are reported, confirm that the following items are present in the relevant location (e.g. figure legend, table legend, main text, or Methods section).

n/a	Confirmed	
☐	☒	The exact sample size (*n*) for each experimental group/condition, given as a discrete number and unit of measurement
☐	☒	An indication of whether measurements were taken from distinct samples or whether the same sample was measured repeatedly
☐	☒	The statistical test(s) used AND whether they are one- or two-sided<br>*Only common tests should be described solely by name; describe more complex techniques in the Methods section.*
☐	☒	A description of all covariates tested
☐	☒	A description of any assumptions or corrections, such as tests of normality and adjustment for multiple comparisons
☐	☒	A full description of the statistics including central tendency (e.g. means) or other basic estimates (e.g. regression coefficient) AND variation (e.g. standard deviation) or associated estimates of uncertainty (e.g. confidence intervals)
☐	☒	For null hypothesis testing, the test statistic (e.g. *F*, *t*, *r*) with confidence intervals, effect sizes, degrees of freedom and *P* value noted<br>*Give P values as exact values whenever suitable.*
☐	☒	For Bayesian analysis, information on the choice of priors and Markov chain Monte Carlo settings
☐	☒	For hierarchical and complex designs, identification of the appropriate level for tests and full reporting of outcomes
☐	☒	Estimates of effect sizes (e.g. Cohen's *d*, Pearson's *r*), indicating how they were calculated
☐	☒	Clearly defined error bars<br>*State explicitly what error bars represent (e.g. SD, SE, CI)*

Our web collection on statistics for biologists may be useful.

Software and code

Policy information about availability of computer code

Data collection	Somatic single-nucleotide mutations identified in whole exome sequencing (WES) studies for 20 tumor types were downloaded from TCGA GDAC Firehose (https://gdac.broadinstitute.org/) (version: analyses__2016_01_28). We obtained the MAF (Mutation Annotation Format, .maf) files by firehose_get (version 0.4.6) (https://confluence.broadinstitute.org/display/GDAC/Download). If several versions of MAF files are obtained for one tumor type (this represents the original MAF file has been filtered against several mutation blacklist files), we took the intersect between MAF files (we only used mutations retained in all MAF files.)
Data analysis	All computations in the present study were performed on a Linux system with multiple Intel E5-2670 2.6GHz, Intel E5-2680 2.4GHz or AMD Opteron 6386 SE processors. The computing environment was managed by conda (v.4.3.27). The exact computing environment (all package info and version) is provided in the bitbucket repo, https://bitbucket.org/szhao06/maps.git, under envs/environment.yaml.

For manuscripts utilizing custom algorithms or software that are central to the research but not yet described in published literature, software must be made available to editors/reviewers upon request. We strongly encourage code deposition in a community repository (e.g. GitHub). See the Nature Research guidelines for submitting code & software for further information.

Data

Policy information about availability of data

All manuscripts must include a data availability statement. This statement should provide the following information, where applicable:

- Accession codes, unique identifiers, or web links for publicly available datasets
- A list of figures that have associated raw data
- A description of any restrictions on data availability

The filtered somatic mutation lists from 20 tumor types that used as input files for driverMAPS and other comparator software are available in Zenodo (DOI: 10.5281/zenodo.1209411).

Field-specific reporting

Please select the best fit for your research. If you are not sure, read the appropriate sections before making your selection.

☒ Life sciences ☐ Behavioural & social sciences ☐ Ecological, evolutionary & environmental sciences

For a reference copy of the document with all sections, see nature.com/authors/policies/ReportingSummary-flat.pdf

Life sciences study design

All studies must disclose on these points even when the disclosure is negative.

Sample size	We used MAF files which were originally aligned to genome build hg19 (GRCh37 Genome Reference Consortium Human Reference 37 (GCA_000001405.1)). This left us with 27 tumor types.
Data exclusions	We excluded THYM (Thymoma) and PCPG (Pheochromocytoma and Paraganglioma) as there weren't suffcient number of mutations (< 2000). We have run driverMAPS for the rest 25 tumor types. Four tumor types, ACC (Adrenocortical carcinoma), LGG (Brain Lower Grade Glioma), THCA (Thyroid carcinoma) and DLBC (Lymphoid Neoplasm Diffuse Large B-cell Lymphoma) have at least 10 novel driver genes identified and the vast majority (>90%) of them were caused by recurrent mutations looking false on reads alignments plots. STAD (Stomach adenocarcinoma) has 111 significantly genes identified and contain >1000 mutations within these genes, making it laborsome to evaluate if the mutations are false or not using reads alignment plots. We excluded these 5 tumor types as the data quality is not ideal or taking too much time to validate. This was done before doing any comparison with other softwares and other downstream analysis.
Replication	For experiments in Fig 6.c-d, Supplementary figure S6 and S7, three independent biological replicates have been performed. All data points are shown on the figure.
Randomization	n/a, this study didn't involve randomazition
Blinding	n/a, blinding is not applicable.

Reporting for specific materials, systems and methods

Materials & experimental systems

n/a	Involved in the study	
☒	☐	Unique biological materials
☒	☐	Antibodies
☐	☒	Eukaryotic cell lines
☒	☐	Palaeontology
☒	☐	Animals and other organisms
☒	☐	Human research participants

Methods

n/a	Involved in the study	
☒	☐	ChIP-seq
☒	☐	Flow cytometry
☒	☐	MRI-based neuroimaging

Eukaryotic cell lines

Policy information about cell lines

Cell line source(s)	The T24 cells used in this study were purchased from ATCC (HTB-4), the 5637 cells used in this study were purchased from ATCC(HTB-9)

Authentication	Authentication was provided by ATCC. As these cell lines were specifically purchased for this project, we didn't perform further authentication procedures.
Mycoplasma contamination	Negative for mycoplasma contamination
Commonly misidentified lines (See ICLAC register)	n/a
